# Characterization of a novel potency endpoint for the evaluation of immune checkpoint blockade in humanized mice

**DOI:** 10.3389/fimmu.2023.1107848

**Published:** 2023-03-03

**Authors:** Alba Matas-Céspedes, Jean-Martin Lapointe, Matthew J. Elder, Gareth J. Browne, Simon J. Dovedi, Lolke de Haan, Shaun Maguire, Richard Stebbings

**Affiliations:** ^1^ Clinical Pharmacology and Safety Sciences, R&D, AstraZeneca, Cambridge, United Kingdom; ^2^ Early Oncology, R&D, AstraZeneca, Cambridge, United Kingdom; ^3^ Antibody Discovery and Protein Engineering, R&D , AstraZeneca, Cambridge, United Kingdom

**Keywords:** humanized mice, immune checkpoint inhibitors, immunotherapy, preclinical safety assessment, GvHD

## Abstract

**Introduction:**

Humanized mice are emerging as valuable models to experimentally evaluate the impact of different immunotherapeutics on the human immune system. These immunodeficient mice are engrafted with human cells or tissues, that then mimic the human immune system, offering an alternative and potentially more predictive preclinical model. Immunodeficient NSG mice engrafted with human CD34+ cord blood stem cells develop human T cells educated against murine MHC. However, autoimmune graft versus host disease (GvHD), mediated by T cells, typically develops 1 year post engraftment.

**Methods:**

Here, we have used the development of GvHD in NSG mice, using donors with HLA alleles predisposed to autoimmunity (psoriasis) to weight in favor of GvHD, as an endpoint to evaluate the relative potency of monoclonal and BiSpecific antibodies targeting PD-1 and CTLA-4 to break immune tolerance.

**Results:**

We found that treatment with either a combination of anti-PD-1 & anti-CTLA-4 mAbs or a quadrivalent anti-PD-1/CTLA-4 BiSpecific (MEDI8500), had enhanced potency compared to treatment with anti-PD-1 or anti-CTLA-4 monotherapies, increasing T cell activity both *in vitro* and *in vivo*. This resulted in accelerated development of GvHD and shorter survival of the humanized mice in these treatment groups commensurate with their on target activity.

**Discussion:**

Our findings demonstrate the potential of humanized mouse models for preclinical evaluation of different immunotherapies and combinations, using acceleration of GvHD development as a surrogate of aggravated antigenic T-cell response against host.

## Introduction

Immunotherapy has emerged in recent years as a novel approach for treatment of cancer through modulation of the patient’s immune system. Antibodies targeting immune checkpoint inhibitors such as programmed cell death-1 (PD-1) its ligand (PD-L1), or cytotoxic T-lymphocyte-associated antigen 4 (CTLA-4) have greatly contributed to advances in cancer treatment in diverse tumour types (1–4). Regulation of immune responses through PD-1 and CTLA-4 is mediated by non-redundant mechanisms (5). While both are negative signals for T cell activation, CTLA-4 is expressed exclusively on T cells and regulates the amount of CD28 co-stimulatory signalling a T cell receives during both activation and also at later stages of differentiation (5, 6). By contrast PD-1 is more broadly expressed and regulates T cells at later stages of immune activation in peripheral tissues or at the tumour site (5). Though recent evidence points to an overlap in the PD-1 and CTLA-4 pathways, given the novel finding that CD80, a ligand for CTLA-4, can dimerise in *cis* with PD-L1 (7). Hence, combined blockade of both immune checkpoints could lead to additive or synergistic effects and improve the response rate to these therapies compared to their use as monotherapies. In this line, clinical data demonstrates the impact of combined therapy with increases in overall survival observed in patients with different solid tumours (8–10).

Syngeneic mouse models are relevant for the study of immunotherapies as they consist of immunocompetent mice engrafted with tumour tissues derived from the same genetic background and can help to understand the immune response after immunotherapy treatment. However, a number of limitations complicate the translational value of these models for humans (11). First, mouse cancer cell lines are limited and do not carry many of the relevant mutations associated with clinical disease. Second, surrogate antibodies, which are functionally equivalent to the therapeutic antibody candidate but binding to the target ortholog expressed in mice, are sometimes difficult to be generated and usually have affinities and binding domains that may not closely resemble the biology of the candidate drug, rendering the data of limited use to understand the pharmacology of the drug on the immune system (11, 12).

Immunodeficient mice engrafted with a functional human immune system are emerging as useful models to evaluate some human immune responses in different pre-clinical scenarios, however they have a number of caveats given their incomplete human immune reconstitution (13–16). The most common immunocompromised strain of mice used for humanization is the NOD-*scid IL2*Rγ^null^ (NSG) (17, 18). These mice can be engrafted with either PBMCs (13) or human haematopoietic stem cells (HSCs), isolated from umbilical cord blood (UCB) (19), bone marrow (BM) (20) or foetal liver (21). This latter model leads to the generation of a diverse repertoire of human immune cells in the mice, including B, T, NK cells, monocytes and dendritic cells, and humanization is improved if newborn mice are used instead of adult mice (22). It typically takes 16 weeks to obtain stable human cell engraftment in mice, and they can survive for more than one year without developing T cells-mediated graft-*versus*-host disease (GvHD). In these humanized mice, injected stem cells are educated against the murine major histocompatibility complex (MHC) during their differentiation into T cells, making them tolerant to the mouse environment, compared to the PBMC model (13), with higher thresholds for their activation (23, 24). The use of human HSCs cells with certain human leucocyte antigen (HLA) types associated with development of autoimmune disorders such as psoriasis or rheumatoid arthritis has been previously reported to accelerate the development of GvHD in this model (25, 26).

In this study, we first compared the *in vitro* biological functionality of different immune checkpoint inhibitors: anti-PD-1 monoclonal antibody (mAb), anti-CTLA-4 mAb, a combination of anti-PD-1 & anti-CTLA-4 mAbs and MEDI8500 that is bivalent for both targets (27) but has reduced affinity to the CTLA-4 receptor. For *in vivo* comparisons, we used UCB-derived CD34^+^ HSCs from donors with HLA alleles predisposed to psoriasis to humanize newborn NSG mice, in order to accelerate development of GvHD, as previously described (25, 26). In these mice we compared the ability of anti-PD-1 and anti-CTLA-4 mAbs as single agents, in combination, and MEDI8500 to break tolerance to the mouse environment, using the development of GvHD lesions as a surrogate to evaluate aggravated antigenic T cell response and as an endpoint to determine relative potency. We demonstrate that both the combination of anti-PD-1 & anti-CTLA-4 mAbs and MEDI8500, show enhanced potency compared to anti-PD-1 or anti-CTLA-4 monotherapy both *in vitro* and *in vivo*. This study provides further evidence for the utility of humanized mice for the evaluation of comparative potency of different immunotherapeutics and combinations.

## Materials and methods

### Antibodies

Anti-PD-1 LO115 is a fully human IgG1 mAb with an affinity for human PD-1 of K_D_ = 0.81nM. Anti-CTLA-4 TM is a fully human IgG1 mAb with an affinity for human CTLA-4 of K_D_ = 0.42nM. Anti-PD-1/CTLA-4 BiS 5 (MEDI8500) is a bivalent BiSpecific antibody where the Fab domains are specific for PD-1, and where the scFv domains are specific for CTLA-4 and are tethered to the CH3 domain. In the BiSpecific format the affinity for human PD-1 is K_D_ = 1.23nM and human CTLA-4 is K_D_ = 1.22nM (**Supplementary Figure 1**). All antibodies were generated by AstraZeneca.

### PD-1 reporter assay

CHO K1 OKT3-CD14 (low) hB7H1 (high) cl 2 cells (expressing anti-CD3 and PD-L1) were seeded at 0.6 × 10^4^ cells in RPMI1640 GlutaMAX™ medium (Gibco, Paisley, UK) supplemented with 10% FBS, in 384-well tissue culture-treated plates and incubated for 18 hours at 37°C. Supernatants were removed and fresh culture medium added with 1.5 × 10^4^ Jurkat NFAT Luc2 PD1 clone 3L-B9 cells (expressing PD-1 and possessing NFAT promoter driven luciferase expression) supplemented with 2.31 nM anti-CD28 antibody (BD Biosciences, Oxford, UK). Single agents anti-PD-1 and anti-CTLA-4 were added to this co-culture in a 3-fold dose titration from 0.005 nM to 300 nM. MEDI8500 was added in a 3-fold dose titration from 0.016 nM to 1000 nM. Co-cultures were incubated for 5 hours and 40 minutes at 37°C followed by 20 minutes at room temperature. Steady-Glo Buffer (Promega, Southampton, UK) was added to tissue culture wells for 10 minutes to lyse cells and luminescence was detected using the using the ultra-sensitive luminescence settings on an Envision spectrophotometer (Perkin Elmer, Seer Green, UK).

### CTLA-4 reporter assay

2 × 10^4^ Raji cells (expressing CD80 and CD86) and 8 × 10^4^ Jurkat CTLA4 IL2 luc2 cells (expressing CTLA-4 and possessing IL-2 promoter driven luciferase expression) were seeded in RPMI1640 GlutaMAX™ medium supplemented with 10% FBS and 1% non-essential amino acids), in 96-well tissue culture-treated plates supplemented with 2.5 µg/mL anti-CD3 (eBioscience, UK). Single agents anti-PD-1 and anti-CTLA-4 were added to this co-culture in a 3-fold dose titration from 0.008 nM to 500 nM. MEDI8500 was added in a 3-fold dose titration from 0.160 nM to 1111 nM. Co-cultures were incubated for 6 hours at 37°C. Steady-Glo Buffer was added to tissue culture wells for 10 minutes to lyse cells and luminescence was detected using the ultra-sensitive luminescence settings on an Envision spectrophotometer.

### Human anti-CD3/SEB assay

Human PBMCs were isolated and re-suspended in assay medium, RPMI1640 GlutaMAX™ medium supplemented with 10% FBS and 1% penicillin-streptomycin and a total of 2 × 10^5^ PBMCs/well were added in triplicates to a 96-well flat-bottomed tissue culture-treated plate, pre-coated for 2 hours at 37°C with 0.5 µg/mL mouse anti-human CD3 antibody (Invitrogen, Paisley, UK), then Staphylococcal enterotoxin B (SEB) (Sigma-Aldrich, St. Louis, MO, USA) was added to a final concentration of 100 ng/mL. Next, anti-PD-1, anti-CTLA-4, MEDI8500, or a combination of anti-PD-1 + anti-CTLA-4 monotherapies were added in a 4-fold dose titration from 400 nM. Cell cultures were incubated at 37°C with 5% CO_2_ for 3 days, supernatants were harvested, and IL-2 secretion was evaluated by ELISA (R&D Systems, Minneapolis, MN, USA).

### Mice

Human CD34^+^ haematopoietic stem cell-engrafted NSG™ females (huNSG) were purchased from The Jackson Laboratory (Sacramento, CA, USA) with specific HLA types predisposed to autoimmunity (**Table 1**). Mice were housed in individually ventilated cages under SPF conditions and used at ~19 weeks of age. Animals were checked daily for morbidity and mortality. At the time of routine monitoring, the animals were checked for any effects of treatments on normal behaviour such as mobility, visual estimation of food and water consumption, body weight gain/loss, eye/hair matting and any other abnormal observations. All study procedures were conducted following an approved IACUC protocol and Crown Bioscience San Diego Standard Operating Procedures.

**Table 1:** HLA profile for each donor.

Donor	2315	2340	5263	5437	5443	5445	5468
**HLA-A**	A*03:02	A*23:01	A*02:01	A*01:03	A*02:01	A*02:01	A*01:01
	A*24:02	A*30:04	A*26:01	A*33:03	A*30:01	A*02:01	A*68:01
**HLA-B**	B*51:01	B*44:03	B*27:05	B*13:02	B*13:02	B*07:02	B*18:01
	B*57:01	B*53:01	B*35:01	B*39:24	B*13:02	B*35:01	B*35:02
**HLA-C**	C*04:01	C*06:02	C*02:02	C*06:02	C*06:02	C*04:01	C*04:01
	C*06:02	C*07:01	C*04:01	C*07:01	C*06:02	C*07:02	C*07:01
**HLA-DRB**	DRB1*04:01	DRB1*07:01	DRB1*04:04	DRB1*11:02	DRB1*07:01	DRB1*11:01	DRB1*08:01
	DRB1*07:01	DRB1*13:04	DRB1*12:01	DRB1*12:01	DRB1*07:01	DRB1*13:05	DRB1*13:01
	DRB4*01:03	DRB3*02:02	DRB3*02:02	DRB3*02:02	DRB4*01:03	DRB3*02:02	DRB3*01:01
	DRB4*01:03N	DRB4*01:01	DRB4*01:03	DRB3*02:02	DRB4*01:03	DRB3*02:02	Blank
**HLA-DQ**	DQB1*03:01	DQB1*02:02	DQB1*03:01	DQB1*03:01	DQB1*02:02	DQB1*03:01	DQB1*04:02
	DQB1*03:03	DQB1*03:01	DQB1*03:02	DQB1*05:01	DQB1*02:02	DQB1*03:01	DQB1*06:03
	DQA1*02:01	DQA1*02:01	DQA1*03:01	DQA1*01:04	DQA1*02:01	DQA1*05:05	DQA1*01:03
	DQA1*03:03	DQA1*05:05	DQA1*05:05	DQA1*05:05	DQA1*02:01	DQA1*05:05	DQA1*04:01
**HLA-DP**	DPB1*04:01G	DPB1*13:01G	DPB1*04:01G	DPB1*01:01G	DPB1*04:01G	DPB1*02:01G	DPB1*02:01G
	DPB1*04:01G	DPB1*17:01G	DPB1*04:01G	DPB1*04:02G	DPB1*20:01G	DPB1*09:01G	DPB1*04:01G
	DPA1*01:03	DPA1*02:01	DPA1*01:03	DPA1*02:02	DPA1*01:03	DPA1*01:03	DPA1*01:03
	DPA1*01:03	DPA1*02:01	DPA1*01:03	DPA1*03:01	DPA1*01:03	DPA1*02:01	DPA1*01:03

The * symbol is a separator used in HLA nomenclature to separate gene and allele group.

### *In vivo* study design

Two different *in vivo* studies were run using the same design. Before grouping and treatment all animals were weighed. The grouping was performed by using StudyDirector™ software (Studylog Systems, Inc. CA, USA). One optimal randomization design (generated by Matched distribution) that showed minimal group to group variation was selected for group allocation. Animals within a single HLA cohort were evenly distributed throughout the groups. Randomization was based on body weight and donor HLA type. Prior to dosing, cheek bleeds were collected from each animal for flow cytometry to detect the baseline immune cell population prior to treatment.

The data presented is a compilation of two different studies. There were 5 treatment groups in total: vehicle (n = 35), anti-PD-1 (n = 15), anti-CTLA-4 (n = 15), combination of anti-PD-1 + anti-CTLA-4 (n = 20) and MEDI8500 (n = 35). Mice received 3 mg/kg of the corresponding single agent or MEDI8500, or a combination of 3mg/kg + 3 mg/kg in the anti-PD-1 + anti-CTLA-4 combination group subcutaneously every 3 days, followed by termination on day 46 (**Supplementary Figure 2**). Body weight and clinical observations were made 3 times a week. Signs and clinical observations of GvHD were established as follows: Grade 0 = Normal; Grade 1 (mild) = 5-10% weight loss, mildly decreased activity, hunching only at rest and/or mild ruffling; Grade 2 (moderate) = 10-20% weight loss, moderately decreased activity, hunching only at rest and/or moderate alopecia; Grade 3 (severe) = >20% weight loss, stationary until stimulated, impaired movement and/or severe ruffling.

Animals were terminated for humane reasons if body weight loss was > 20% over a period of 72 hours. Upon termination, blood was collected for plasma processing and flow cytometric analysis. Lungs, liver and half of the spleen were collected for histology and immunohistochemistry. The other half of the spleen was processed for flow cytometric analysis. For any animals found dead, tissues were not taken for analysis.

### Histopathology and immunohistochemistry

Samples of liver, lung and spleen were fixed in 10% neutral-buffered formalin, and processed to paraffin blocks using routine methods. Previous observations (unpublished) had shown that these organs were the most consistently and strongly affected in animals that developed GvHD. Tissues were sectioned at 4 μm thickness and stained with hematoxylin and eosin (H&E), or with immunohistochemistry using a rabbit monoclonal antibody specific for human CD45 (D9M81, Cell Signaling Technology, Leiden, The Netherlands) at a 0.05 µg/mL dilution, on an automated Leica Bond-RX immunostainer (Leica Biosystems, Wetzlar, Germany), using DAB as a chromogen. This antibody was demonstrated not to cross-react with mouse CD45 in wild-type mouse lymphoid tissues (unpublished observations).

For histopathology evaluation, H&E stained sections were examined by a board-certified veterinary pathologist with experience in mouse pathology, and evaluated for inflammatory/immune changes. Changes that were considered by the pathologist to be a consequence of GvHD (rather than background/incidental changes) were characterised and scored for severity on a subjective scale of 1 to 4, depending on the extent of the change thoughout the tissue. A total GvHD score (0-12) was calculated by adding the lesion scores from each tissue (if more than one change was observed per tissue, only the highest lesion score was used for that tissue).

For immunohistochemistry evaluation, sections stained for huCD45 were digitally scanned at 20x magnification, using an Aperio scanner (Leica Biosystems). The extent of huCD45 cell infiltration in the tissues was quantified using Halo image analysis software (Indica Labs, Albuquerque, NM, USA). Briefly, the tissue region of interest (ROI) was annotated by the pathologist, targeting the maximal amount of tissue, but excluding large tissue artifacts (debris, folds, etc…) and extraneous tissue. Positive huCD45 staining was quantified, using the Halo Area Quantification algorithm (v.2.1.3), adapted to the staining characteristics of the study. An area-based detection was considered more representative than cell-based quantification, because the intensity of huCD45 staining and the tendency of huCD45-positive cells to form dense clusters made single-cell recognition difficult. The huCD45-positive area was reported as percentage of total tissue area (excluding clear spaces).

### Flow cytometry

Flow cytometric analysis was conducted on whole blood from cheek bleeds prior to dosing, and also on blood and spleen from all mice taken at termination, except for those found dead. Upon collection of blood samples, red blood cells (RBC) were lysed first with 1x RBC Lysis Buffer (Invitrogen, Waltham, MA, USA), followed by mouse Fc blocking with TruStain FcX™ (anti-mouse CD16/32, BioLegend, San Diego, CA, USA) and human Fc blocking with Human TruStain FcX™ (BioLegend) prior to the staining. The spleens were smashed through PBS pre-wet 70-μM cell strainers, followed by RBC lysis and Fc blocking before staining. Single cells were stained with the following anti-human antibodies obtained from BioLegend: CD45 (clone 2D1), CD3 (clone HIT3a), CD8 (clone SK1), CD4 (clone RPA-T4), CD154 (CD40L) (clone 24-31) and CD134 (OX40) (clone Ber-ACT35). Stained samples were run on a BD LSRFortessa™ flow cytometer (BD Biosciences, San Jose, CA, USA). Data were analysed with the Kaluza Analysis Software (Beckman Coulter, Brea, CA, USA). Human cells were phenotyped following the gating strategy presented in **Supplementary Figure 3**.

### Statistical analysis

Data analysis was conducted using GraphPad Prism 8 (GraphPad Software, San Diego, CA, USA). Survival curves were represented with Kaplan-Meier plots and statistical differences calculated using the Mantel-Cox test. A Mixed-effects analysis followed by Sidak’s multiple comparisons test was used to evaluate statistical differences between pre-treatment and post-treatment groups. A one-way ANOVA followed by Tukey’s multiple comparison was used to evaluate statistical differences between treatment and control groups. Differences in incidence of histologic GvHD lesions between groups were evaluated with chi-square tests.

## Results

### *In vitro* functionality of monoclonal antibodies and MEDI8500 targeting PD-1 and CTLA-4

**Figure 1:**
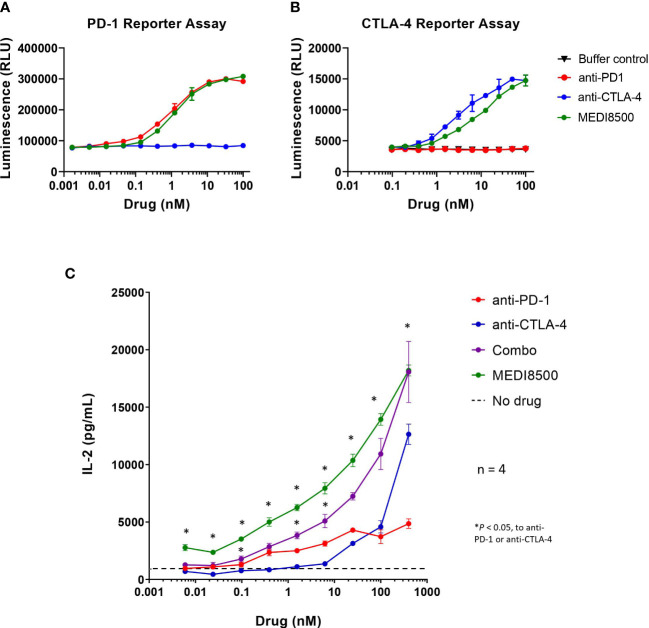
Increased *in vitro* potency with antibody combination and MEDI8500 compared to monotherapies **(A)** Bioluminescence levels in a PD-1/PD-L1 blockade bioassay after 5 hours and 40 minutes of incubation with increasing doses (3-fold) of either anti-PD-1, anti-CTLA-4 or MEDI8500. **(B)** Bioluminescence levels in a CTLA-4 blockade bioassay after 6 hours of incubation with increasing doses (3-fold) of either anti-PD-1, anti-CTLA-4 or MEDI8500. Data are represented as Mean ± SD of duplicates in each point. **(C)** Levels of IL-2 secretion by human PBMCs after 72h of *in vitro* treatment with increasing doses (4-fold) of either anti-PD-1, anti-CTLA-4, combination of anti-PD-1 + anti-CTLA-4 or MEDI8500, on an anti-huCD3 + SEB stimulation assay. Data are represented as Mean ± SEM for 4 independent donors. A two-way ANOVA followed by Tukey’s multiple comparison was used to evaluate statistical differences between antibody combination or MEDI8500 and single agents at each concentration; statistically significant differences are noted when *P < 0.05.

We performed two separate reporter assays to evaluate the *in vitro* potency of the different antibodies blocking both pathways: PD-1/PD-L1 and CTLA-4/CD80&86. In the PD-1 reporter assay, we observed that the potency for human PD-1 reporter blockade of anti-PD-1 mAb as single agent (EC50 = 0.92 nM) was similar to that of MEDI8500 (EC50 = 1.30 nM) (**Figure 1A**). In the CTLA-4 reporter assay, the potency for human CTLA-4 receptor blockade of anti-CTLA-4 mAb as single agent (EC50 = 3.57 nM) was ~ 4-fold higher compared to that of MEDI8500 (EC50 = 12.61 nM) (**Figure 1B**).

Moreover, treatment of primary PBMCs with MEDI8500 significantly increased the levels of IL-2 secreted by PBMCs at all concentrations after 72 hours, compared to the respective single agents (**Figure 1C**). Similarly, the combination of anti-PD-1 + anti-CTLA-4 mAbs significantly raised the levels of IL-2 secreted at different concentrations compared to the single agents, but to a lesser extent than MEDI8500. No significant differences were observed in the level of IL-2 secretion between MEDI8500 and combination treatment (**Figure 1C**).

### *In vivo* functionality of monoclonal antibodies and MEDI8500 targeting PD-1 and CTLA-4

**Figure 2:**
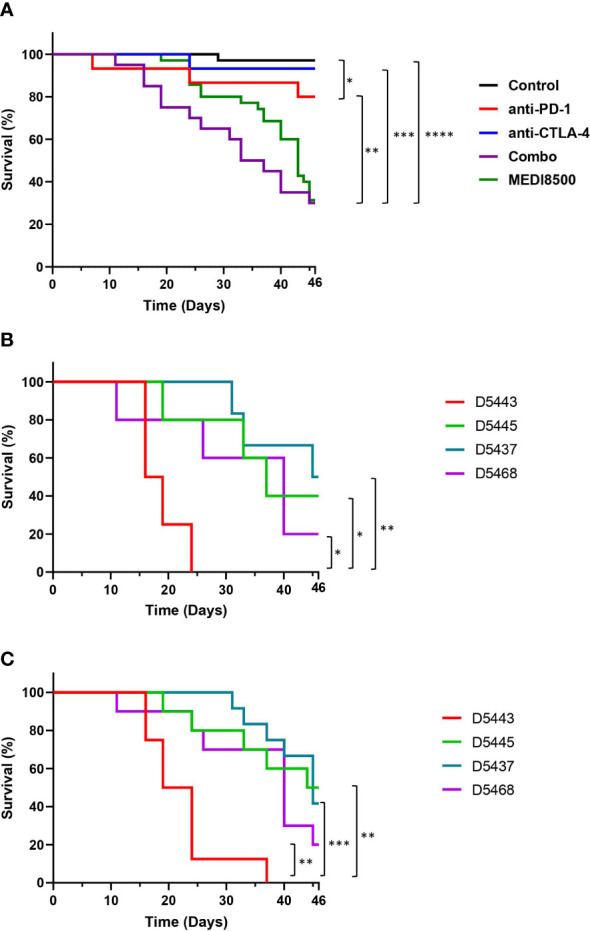
Acceleration of GvHD with antibody combination and MEDI8500 compared to monotherapies **(A)** Kaplan-Meier plot showing overall survival in the different groups of treatment. The control (n=35) and MEDI8500 (n=35) groups include data from two independent experiments, while the data for the anti-PD-1 (n=15), anti-CTLA-4 (n=15) and antibody combination (n=20) groups are from a single experiment. **(B)** Survival curves in the antibody combination group depending on the HLA type of the donor. Each donor showed was used to reconstitute 4-6 mice per treatment group. **(C)** Survival curves in MEDI8500 group depending on the HLA type of the donor. Each donor showed was used to reconstitute 4-6 mice per treatment group. The Mantel-cox test was used to assess statistical differences between groups; *P < 0.05, **P < 0.01, ***P < 0.001, ****P < 0.0001.

Acceleration of GvHD in huNSG mice after immunotherapy treatment was used as an endpoint to evaluate the relative potency of monoclonal & BiSpecific antibodies targeting PD-1 and CTLA-4. Most animals treated with the single agents did not develop symptoms of GvHD (Grade 0) or had mild signs of rough coats and hunching (Grade 0-1) between day 24-46, and the majority survived until the end of the study (12 out of 15 in the anti-PD-1 mAb group and 14 out of 15 in the anti-CTLA-4 mAb group). However, survival was significantly decreased in the anti-PD-1 group compared to vehicle (**Figure 2A**). In the mice that received the anti-PD-1 + anti-CTLA-4 combination significantly reduced survival was noted compared to the control group, and when compared to the anti-PD-1 or anti-CTLA-4 single agent groups (**Figure 2A**), with some animals terminated as soon as 11 days after the start of treatment due to the onset of Grade 3 adverse signs, including moderate rough coats and hunching, in addition to weight loss (**Supplementary Figure 4**), and just 6 out of 20 mice (30%) surviving until the end of the study with mild (Grade 0-1) or moderate (Grade 2) symptoms of GvHD. Similarly, mice in the MEDI8500 group showed a significantly decreased survival compared to the control animals, and animals receiving anti-PD-1 or anti-CTLA-4 as single agents, with mice terminated from day 19 onwards due to development of Grade 3 GvHD phenotype, including moderate rough coats, hunching and emaciation. Just 11 out of 35 mice (31%) survived until the end of the study with mild (Grade 0-1) or moderate (Grade 2) symptoms of GvHD. There was no significant difference in survival between the antibody combination and MEDI8500 groups.

We observed differences in survival rate after treatment between some of the donor HLA types in both the antibody combination (**Figure 2B**) and MEDI8500 group (**Figure 2C**). Mice reconstituted with cells from donor 5443 showed a significant reduction in overall survival after treatment with either the antibody combination or MEDI8500, with all mice terminated due to adverse GvHD signs Grade 3 between days 16-24 after the start of treatment in the antibody combination group (**Figure 2B**) or after 16-37 days in MEDI8500 group (**Figure 2C**). Donor 5443 was unusual, in that this donor was homozygous for a total of 7 out of 9 HLA alleles: HLA-B, HLA-C, HLA-DRB1, DRB4, DQA1, DQB1 and DPA1, with specific HLA alleles more predisposed to autoimmunity (**Table 1**). By contrast, the other donors were more heterozygous, with at most 4 homozygous alleles, and mice reconstituted with stem cells from more heterozygous donors survived significantly longer with both treatment regimens (**Figures 2B, C**).

### Expansion of T cells in humanized mice after administration of PD-1 and CTLA-4 targeting antibodies

**Figure 3:**
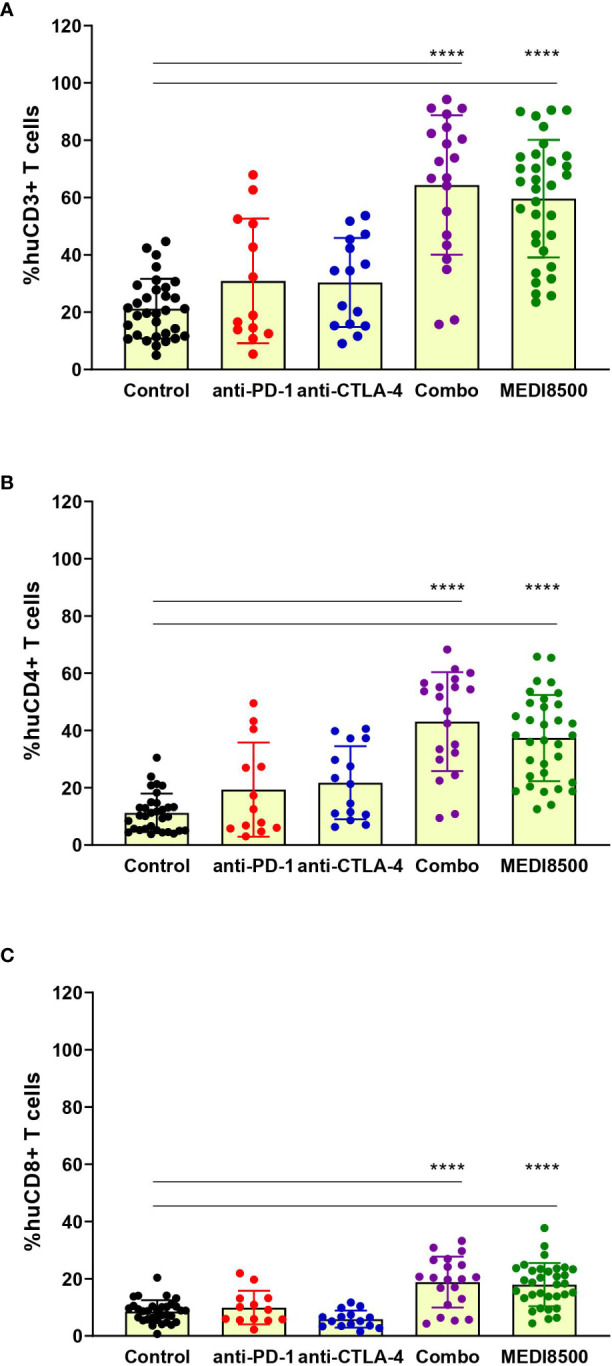
Significant expansion of T cells with antibody combination and MEDI8500 compared to monotherapies. Flow cytometry analysis of the percentages of **(A)** huCD3^+^ T cells, **(B)** huCD4^+^ T cells and **(C)** huCD8^+^ T cells in spleen of mice at sacrifice. Data are represented as percentage within huCD45^+^ population for each individual mouse and as Mean ± SD of all mice in each treatment group. Mice numbers are the same as in **Figure 2A**. A one-way ANOVA followed by Tukey’s multiple comparison was used to evaluate statistical differences between treatment and control groups; ****P < 0.0001.

Analysis of the percentage of huCD3^+^ T cell population in dissociated spleen at termination revealed significant T cell expansion in the antibody combination (> 60%) and MEDI8500 (> 55%) groups compared to the control group (~ 20%) and the single agent groups (~ 30%) (**Figure 3A**). Similarly, the percentages of huCD4^+^ and huCD8^+^ T cells were significantly increased solely in the antibody combination and MEDI8500 groups (huCD4^+^ > 35%; huCD8^+^ ~ 18%) compared to the control (~ 10%) and single agent groups (**Figures 3B, C**).

Likewise, a significant increase on the percentage of huCD3^+^ T cell population was found in blood at termination in the different groups after treatment compared to the pre-treatment levels (**Supplementary Figure 5A**). This rise in huCD3^+^ T cells corresponded with a significant increase of circulating huCD4^+^ and huCD8^+^ T cells in the antibody combination and MEDI8500 groups after treatment (huCD4^+^ > 50%; huCD8^+^ > 20%), when compared to the control and anti-PD-1 groups (**Supplementary Figures 5B, C**). The percentage of huCD4^+^ T cells was also significantly higher in the anti-CTLA-4 single agent group (> 35%) after treatment (**Supplementary Figure 5B**), but not the percentage of huCD8^+^ T cells (**Supplementary Figure 5C**).

### Activation of T cells in humanized mice after administration of PD-1 and CTLA-4 targeting antibodies

**Figure 4:**
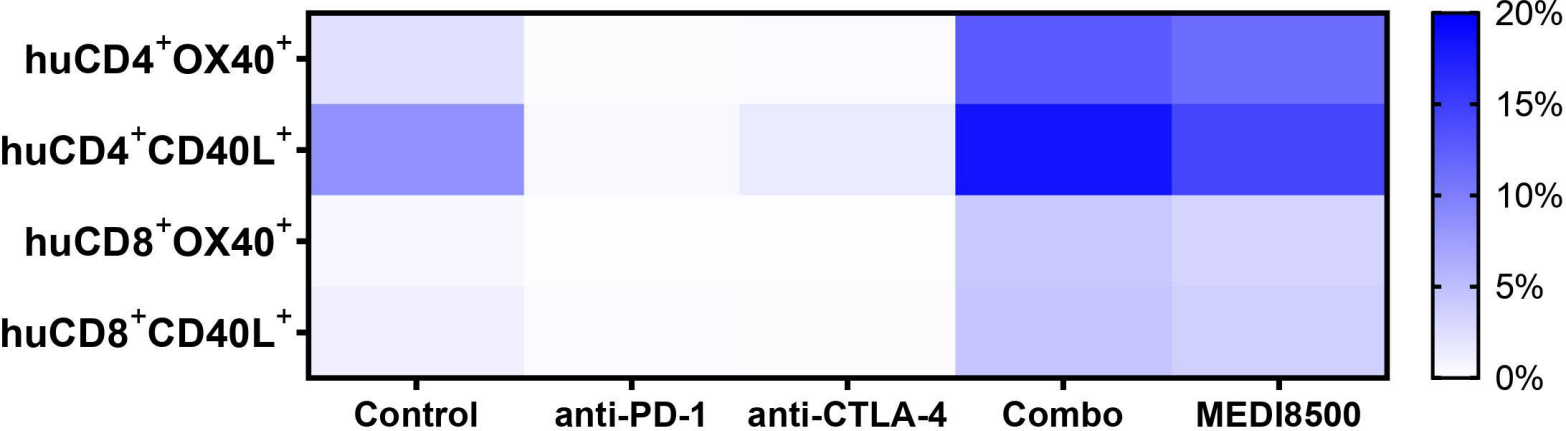
Significant activation of T cells with antibody combination and MEDI8500 compared to monotherapies. Percentage of huCD4^+^ and huCD8^+^ T cells expressing T cell activation markers in terminal blood of mice in the different groups of treatment. Data were analysed by flow cytometry and represented as mean of all mice in each treatment group. Mice numbers are the same as in **Figure 2A**. A Mixed-effects analysis followed by Dunnett’s multiple comparisons test was used to evaluate statistical differences between control and treated groups. A significant difference (***P < 0.001) was noted in the antibody combination and MEDI8500 groups compared to the control group in the percentage of huCD4^+^OX40^+^ T cells, huCD8^+^OX40^+^ T cells and huCD8^+^CD40L^+^ T cells. *P < 0.05 was noted in the antibody combination and MEDI8500 groups compared to the control group in the percentage of huCD4^+^CD40L^+^ T cells.

We evaluated the expression levels of different activation markers on human T cells circulating in blood in the different groups of mice at sacrifice. We observed that T cells from mice from the antibody combination and MEDI8500 groups showed a significantly increased percentage of OX40^+^ ( ~ 13% and ~ 11%, respectively) and CD40L^+^ ( ~ 18% and ~ 14%) huCD4^+^ T cells compared to the control group (**Figure 4**). Similarly, we found a significant increase of huCD8^+^ T cells expressing these markers in the antibody combination and MEDI8500 groups (OX40^+^ ~ 4% and ~ 3%, respectively; CD40L^+^ ~ 4% and ~ 3%, respectively) when compared to control, but the extent of increase in the percentage of cells expressing these markers was lower on huCD8+ T cells than on huCD4^+^ T cells (**Figure 4**).

### Highest incidence of GvHD lesions after antibody combination or MEDI8500 in humanized mice

**Figure 5:**
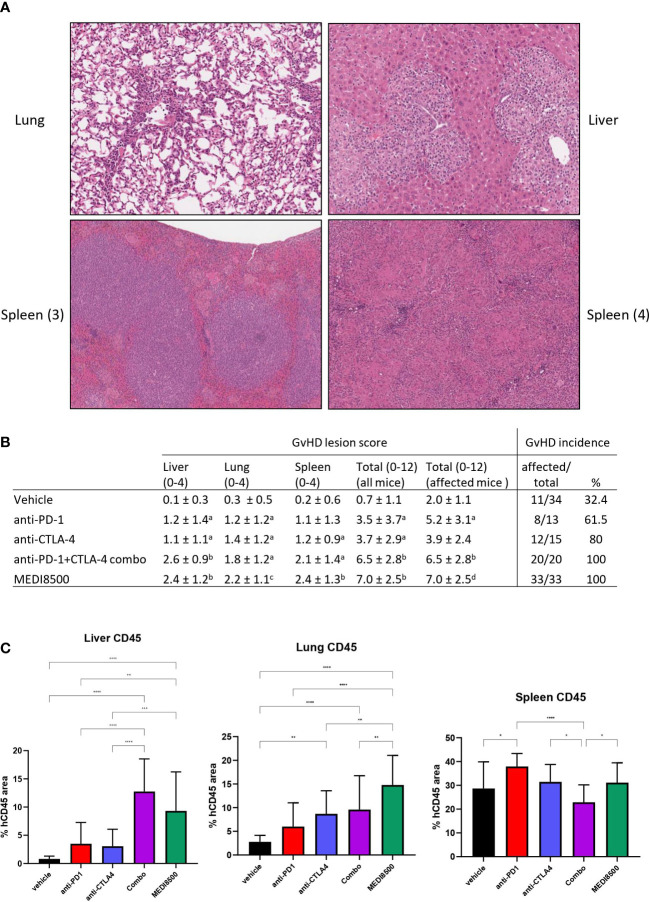
Greater GvHD lesion score with antibody combination and MEDI8500 compared to monotherapies. **(A)** Representative images of GvHD lesions in tissues: (1) lung, with vascular intimal, perivascular and interstitial lymphocytic infiltration (20x magnification) (2) Liver, with portal infiltration with lymphocytes and macrophages extending to the lobular parenchyma (20x magnification) (3) spleen, with expansion of white pulp by lymphocytes, and small macrophage nodules in the red pulp (10x magnification) (4) spleen, with diffuse granulomatous infiltration with coalescing macrophage nodules (12x magnification) **(B)** Histopathologic scoring of GvHD lesions, shown as mean ± SD per treatment group. Total score is sum of liver, lung and spleen scores. Mean total scores are calculated either for the whole group (including animals without any lesions), or excluding the animals without lesions. ^a^ significant difference (*P < 0.05) with vehicle, ^b^ significant difference (*P < 0.05) with vehicle, anti-PD-1 and anti-CTLA-4, ^c^ significant difference (*P < 0.05) with vehicle and anti-PD-1, ^d^ significant difference (*P < 0.05) with vehicle and anti-CTLA-4 **(C)** huCD45 positive cell infiltration in liver, lung and spleen, expressed as proportion of total tissue area. **P<0.01, ***P<0.001, ****P<0.0001.

Sections of liver, lung and spleen stained with H&E were examined to detect inflammatory changes consistent with GvHD (**Figure 5**). In the liver (**Figure 5A**), inflammatory changes consisted mostly of infiltration of the portal spaces by immune cells, predominantly lymphocytes but frequently accompanied by macrophages. This infiltrate often breached the portal limiting plate to extend into the lobular parenchyma, and was occasionally associated with the presence of single necrotic hepatocytes within the infiltrate. This immune infiltrate was also occasionally observed perivascularly, around centrolobular veins, as well as in random foci in the lobular parenchyma. In the more severe cases the immune infiltrate was very abundant, with miliary foci throughout the tissue. In some cases the infiltrate had a distinctly granulomatous component, with prominent macrophages and multinucleated giant cells.

In the lung (**Figure 5A**), the observed inflammatory changes consisted predominantly of perivascular immune cell infiltrates around small and medium sized vessels, sometimes associated with leukocyte margination in the vascular lumen and focal intimal immune cell infiltration. The immune cells were predominantly lymphocytes, with the macrophage component observed in the liver not as prominent in the lung lesion. The alveolar parenchyma around the vessels with perivascular infiltrates often contained small foci of interstitial immune cell infiltration. In the more severe cases, the perivascular infiltrates formed thick cuffs, with the intima markedly thickened by immune cells, and the surrounding parenchyma showing significant interstitial infiltration.

In the spleen (**Figure 5A**), the changes were variable. Expansion of the periarteriolar white pulp areas by a dense population of immune cells, mostly lymphocytes, was frequently observed, but in some cases significant numbers of macrophages were also present. Another frequently observed change was infiltration of the red pulp area with small nodules of macrophages and occasional multinucleate giant cells; in more severe cases this infiltration became almost diffuse throughout the red pulp, significantly expanding the spleen, and associated with areas of necrosis. Often, a significant and variable degree of extra-medullary hematopoiesis (EMH) was observed. This is a common observation in mouse spleen, and this was not interpreted as associated with GvHD. However, individual variability in EMH likely had a confounding effect on relative huCD45^+^ area measurements.

The incidence of mice with GvHD lesions in at least one tissue and the severity score of GvHD lesions per group are shown in **Figure 5B**. The incidence of histologic lesions was clearly higher than the incidence of pre-mortem clinical signs. Overall, there were more frequent GvHD lesions in the anti-PD-1 and anti-CTLA-4 groups than in the vehicle group, and more frequent lesions in the antibody combination and MEDI8500 groups than in the vehicle and monotherapy groups. A similar pattern was noted in the total lesion severity scores, with the antibody combination and MEDI8500 groups showing higher mean scores than the monotherapy groups, and the latter groups showing higher severity scores than the vehicle group. This overall pattern was preserved when animals without lesions were excluded. This indicates that, when present, lesions tended to be more severe in the antibody combination and MEDI8500 groups than in the monotherapy and vehicle groups. In the monotherapy groups, when present, lesions were more severe in the anti-PD-1 group than in the vehicle group, but there was no statistically significant difference in lesion severity between the CTLA-4 group and vehicle. Finally, there were no differences in lesion incidence or overall severity between the antibody combination and MEDI8500 groups. Potential effect of donors on the incidence and severity of GvHD lesions was difficult to reliably assess, because of the small number of animals per donor within each treatment group, and the individual variability in GvHD lesion scores.

Immunohistochemical staining of liver, lung and spleen section with a huCD45-specific antibody was carried out in an attempt to quantify GvHD-linked immune cell infiltration, and compare these results to histopathologic evaluation (**Figure 5C** and **Supplementary Figure 6**). Significant increases in huCD45 staining were noted in the livers of some treatment groups, mainly the antibody combination and MEDI8500 groups which showed significantly higher huCD45 infiltration than the vehicle and monotherapy groups. In the lung, the anti-CTLA-4, antibody combination and MEDI8500 groups showed higher huCD45 staining than the vehicle group, with MEDI8500 group showing the most marked increase in huCD45 staining. These results tended to mirror the results from the GvHD lesion scores, although GvHD lesion scores tended to show more statistically significant differences between groups than huCD45 staining (**Supplementary Table 1**). In the spleen, some differences in huCD45 staining were noted between groups, but these did not correlate with GvHD lesion severity, and therefore huCD45 staining was not helpful in quantifying GvHD lesions in the spleen (**Supplementary Figure 6**).

## Discussion

In this study we have analysed the *in vitro* functionality of the immune checkpoint inhibitors anti-PD-1 and anti-CTLA-4 antibodies as either single agents or in combination, along with MEDI8500, which is an anti-PD-1/CTLA-4 BiSpecific antibody. PD-1 and CTLA-4 are immune checkpoint inhibitors that provide negative signals for T cell activation after interaction with their ligands, helping to maintain immune tolerance (5). In the clinic, when anti-PD-1 and anti-CTLA-4 mAbs are administered as monotherapy, patients who respond can have significantly increased survival. However, only a proportion of patients respond to these monotherapies (28), hence there was rationale for combining PD-1 and CTLA-4 inhibitors, as it could lead to synergistic increases in T cell activation and patient response rate (28). The combination of anti-PD-1 and anti-CTLA-4 mAbs has shown improved responses in the clinic and a sustained survival benefit, compared to those in the monotherapy groups (8–10, 29–31), and is approved for different types of solid cancers (32). However these combinations can lead to an increase in immune related adverse events. Development of an anti-PD-1/CTLA-4 BiSpecific antibody is a novel approach aiming to release the full potential of the combination into a single molecule. MEDI8500 has a similar potency against human PD-1 as the anti-PD-1 mAb, but it has a lower affinity for the CTLA-4 receptor and a 4-fold lower potency than the anti-CTLA-4 mAb. The first-in-class anti-PD-1/CTLA-4 bispecific to be recently approved for the treatment of advanced cervical cancer has been Cadonilimab (33), which is a quadrivalent bispecific with similar format to MEDI8500. Similarly, MEDI5752, which is a monovalent anti-PD-1/CTLA-4 BiSpecific (34), is currently under evaluation in patients and durable responses have been seen across diverse tumour types (35–37). In this line, our *in vitro* data demonstrate that both the anti-PD-1 and anti-CTLA-4 combination and MEDI8500 induced increased activity on primary immune cells compared to the single agents treatment.

We next evaluated the *in vivo* potency of anti-PD-1 and anti-CTLA-4 mAbs as monotherapies, combination and MEDI8500 in non tumour-bearing humanized mice, using GvHD development as a potential predictor of toxicity and potency of these immunotherapies and combinations. Our model used mice reconstituted with CD34^+^ HSCs cells derived from donors with HLA alleles predisposed to autoimmune disorders. The use of HLA donors predisposed to autoimmunity has previously been described for studies of the pathogenesis of GvHD (25, 26). For our purposes, use of such donors was designed to increase the incidence of GvHD after treatment with immunotherapies and potentially shorten the study duration. However, for other study purposes these HLA type donors should be avoided in order to lengthen the survival of the humanized mice.

As expected, we observed a significantly lower survival rate due to accelerated development of GvHD in the checkpoint inhibitor treated groups compared to the controls, consistent with enhanced immune activation in humanized mice treated with these agents. The anti-PD-1 + anti-CTLA-4 combination and MEDI8500 groups showed lower survival, and accelerated onset of clinical GvHD compared to vehicle and to the single agents anti-PD-1 and anti-CTLA-4, and generally higher GvHD lesion levels compared to the single agent treated mice.

Certain HLA types are more likely to be susceptible to autoimmune disorders, viral infections or development of cancer (26, 38). Patients with autoimmune disorders treated with immune checkpoint inhibitors may show aggravation of their disease, more severely when given in combination (39). In our study, there was evidence of an effect of the donor HLA type on the onset and severity of GvHD reactions. Specifically, mice reconstituted with cells from one homozygous HLA type donor (D5443) appeared to have lower survival rate and acceleration of GvHD development compared with mice reconstituted with HLA donors with higher heterozygosity, in response to anti-PD-1 + anti-CTLA-4 combination and MEDI8500.

Flow cytometric analysis of circulating human lymphocyte profiles in the mice revealed a significant increase in the percentage of huCD3^+^ T cells in all groups, including the controls. This increase in controls was likely due to a baseline immune activation associated with incipient development of GvHD, as was seen histologically in several of the control animals. The choice of human cell donors predisposed to autoimmunity would be expected to lead to quicker development of GvHD reactions even in the absence of immune stimulatory drugs, and therefore this should be carefully considered when interpreting data from this model; effect of administered drugs should be interpreted in light of the baseline immune activation present. This was the case in this study, where we saw a significant increase in the percentage of huCD3^+^, huCD4^+^ and huCD8^+^ T cells in groups treated with either the anti-PD-1 + anti-CTLA-4 combination or MEDI8500, compared to the control or anti-PD-1 or anti-CTLA-4 monotherapy groups. PD-1 and CTLA-4 act at different stages of T cell activation, thus, combined blockade of both immune checkpoint inhibitors has synergistic effects on T cell activation and proliferation, as aforementioned (28). The increase in percentage of circulating human lymphocytes has been reported previously after treatment of humanized mice with either anti-PD-1 or anti-CTLA-4 mAbs (40, 41), and it likely reflects an increased production of these cells as a response to induction of GvHD reactions in various tissues by PD-1 and CTLA-4 blockade. Furthermore, it was also associated with an increase in T cell activation. We used 2 different cell markers associated with T cell activation: OX40, which is not constitutively expressed on resting naïve T cells and expressed after 24 to 72 hours following activation (42); and CD40L, which is predominantly expressed by activated huCD4^+^ T cells shortly after T cell activation (43). We observed a significant increase in activated T cells in terminal blood with antibody combination and MEDI8500 treatments, compared to vehicle and monotherapy groups.

Histologic evaluation of liver, lung and spleen confirmed the presence of GvHD type lesions in these mice. This evaluation not only revealed lesions in mice culled prematurely due to adverse clinical signs, but also in mice that survived until the end of the study. However mice that were culled prematurely showed more severe lesions, an increased GvHD score and greater influx of huCD45+ based on IHC analysis.

Although some of the control group animals had GvHD lesions, these were mild, and not associated with adverse clinical signs or body weight loss (**Supplementary Figure 4**). This early development of GvHD was likely related to the use of donors predisposed to autoimmunity, and there was some evidence for donor-related differences in the incidence of lesions. The presence of such lesions underscores the need for careful study design when using such a model, with a need to use multiple donors and distribute donors evenly throughout the treatment groups to avoid confounding effects of variable degrees of donor predisposition to GvHD.

The histopathological lesions observed in these mice were consistent with those previously reported for humanized mice (14, 44) and with some of the changes reported for chronic human GvHD (45, 46), although overall the mouse lesions tended to be less variable than the fairly broad spectrum of changes reported in humans. This is not surprising, considering the consistent and well-controlled methodology, limited genetic variability of the host mice, and use of a limited pool of donors in this model compared to the myriad possible variations in the human population. One frequent feature of the mouse lesions was the presence of a significant, often abundant, macrophage component within the immune cell infiltrate of the liver or spleen; in some cases macrophages were the dominant immune population, forming granulomatous nodular infiltrates. This has also been previously reported in this model (14), but it is unclear why this cell population becomes stimulated to this extent, and why it is prominent in some mice and not others. There did not appear to be an association of this observation with specific treatments or donors.

In order to be able to compare different treatments based on histopathology, we evaluated the incidence of GvHD lesions per group, and developed a semi-quantitative histopathologic scoring system based on lesion severity. Both incidence and severity showed clear differences between groups, with the severity score providing more granularity to the analysis, and better differentiation between groups. When considering the total severity score (combined scores for the liver, lung and spleen), significant differences were evident between the anti-PD-1 or anti-CTLA-4 monotherapy groups and the control group, as well as between the antibody combination or MEDI8500 groups and the monotherapy and vehicle groups. This confirmed the additive immune-stimulatory activity of anti-PD-1 and and-CTLA-4, when combined as single agents or as a BiSpecific. We could not detect clear differences in activity between the antibody combination and MEDI8500 treatment.

Immunohistochemical staining for huCD45 was applied to the liver, lung and spleen, with image analysis quantitation of positive cells, in an effort to develop a quantitative tool for GvHD evaluation. This proved a useful way to obtain more quantitative and objective data on human immune cell infiltration in liver and lung, with huCD45 positivity generally mirrorring the GvHD lesion scores; however it proved to be somewhat less sensitive than histopathologic evaluation in identifying the early, milder lesions. This is most likely because immune cell infiltration in GvHD lesions must attain a threshold in order to be detectable above of the normal baseline level of huCD45 cells in tissues, which are either circulating through the vasculature or resident tissue immune cells. In line with this notion, liver and lung huCD45 staining in the antibody combination and MEDI8500 groups, which had higher lesion severity scores, clearly was significantly increased when compared to controls and monotherapy groups, whilst huCD45 staining in monotherapy groups were generally not high enough to show statistically significant differences from controls, despite presence of mild histopathologic lesions. In the spleen, huCD45 staining did not consistently detect GvHD lesions. This was likely due to the fact that human cells administered to these mice would be expected to home in to normal lymphoid tissue sites like the spleen, and therefore a large proportion of cells within the spleen of reconstituted mice would normally be huCD45^+^ in the absence of any GvHD reaction.

In summary, evaluation of mice with a humanized immune system showed it was a useful model for comparing the *in vivo* potency and safety profile of PD-1 and CTLA-4 based immuno-oncology therapeutics. Analysis of blood and tissue responses provided quantitative and semi-quantitative data with sufficient granularity to differentiate the potency of different treatment modalities. Induction of GvHD in these mice was a consistent endpoint for demonstration of immunotherapeutic potency. This aggravated antigenic T-cell response against host due to checkpoint blockade is a surrogate of the antigenic T-cell response that can be potent in targeting cancer cells or any other foreign antigens. Histopathologic evaluation of liver, lung and spleen was the most sensitive indicator of GvHD, compared to huCD45 IHC or clinical observations. Administration of anti-PD-1 or anti-CTLA-4 as single agents induced a significant degree of immune stimulation, but significantly less than a combination of the two or MEDI8500. However, the results in the monotherapy groups do not completely correlate with the safety profile of anti-CTLA-4 mAbs in the clinic, where these therapies have much more severe immune mediated adverse reactions than anti-PD-1 mAbs when given as monotherapies (47). Taken together, the use of humanized mice may be a useful platform to evaluate the function of human immunotherapies, where no other preclinical models exist. These models may also provide information on the potential for immune related adverse reactions with immunotherapies combination, but like all animal models, this model has limitations which must be understood and improved upon to increase their translational predictivity to the clinical situation.

## Data availability statement

The original contributions presented in the study are included in the article/**Supplementary Material**. Further inquiries can be directed to the corresponding authors.

## Ethics statement

The animal study was reviewed and approved by AstraZeneca ethics review.

## Author contributions

RS, SM and LH contributed to conception and design of the *in vivo* study. J-ML performed the histology evaluation. ME and GB performed the *in vitro* experiments. SD surpevised the *in vitro* experiments and project development. AM-C performed data analysis and interpretation. AM-C wrote the first draft of the manuscript. J-ML and RS wrote sections of the manuscript. All authors contributed to manuscript revision, read, and approved the submitted version.
