# Supplementary material for: Characterization of a novel potency endpoint for the evaluation of immune checkpoint blockade in humanized mice
Source: Front Immunol. 2023 Mar 3;14:1107848. doi: 10.3389/fimmu.2023.1107848 (PMC10020612; doi:10.3389/fimmu.2023.1107848)
Supplement: Supplementary file 1 [file DataSheet_1.pdf]

<u>Supp figure 1</u>: Monoclonal antibody formats

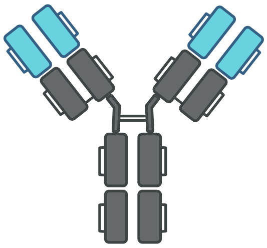

Bivalent IgG1

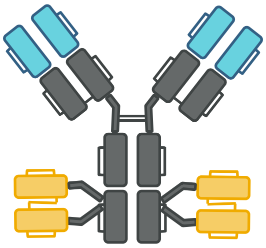

MEDI8500

Anti-PD-1 LO115 and anti-CTLA-4 TM are bivalent fully human IgG1 monoclonal antibodies (mAbs) (left molecule), whereas MEDI8500 is a bivalent bispecific mAb with 2 scFv attached to the middle of the Fc (right molecule).

<u>Supp figure 2</u>: *In vivo* experimental design

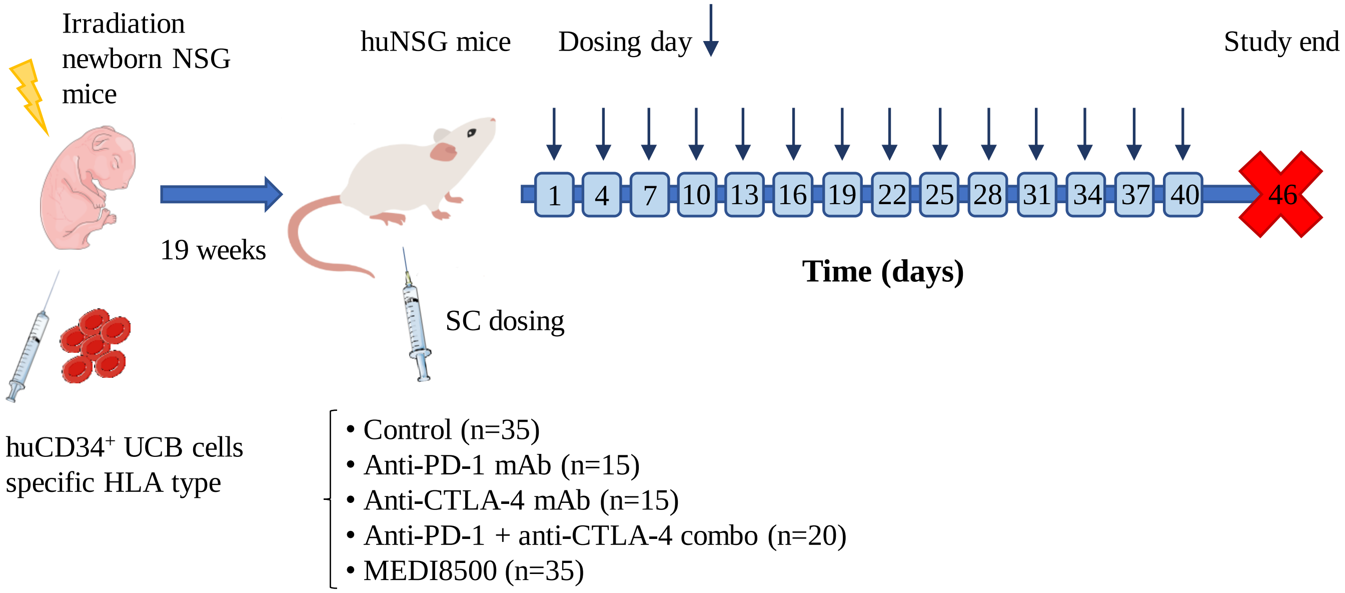

Newborn NSG females were irradiated and subsequently injected with huCD34+ UCB derived cells with specific HLA types predisposed to autoimmunity. After 19 weeks of humanization, mice were randomized and distributed in 5 groups of treatment. Mice were subcutaneously (SC) dosed every 3 days with 3mg/kg of each mAb. Animals were sacrificed when they developed symptoms of GvHD, or at study end on day 46.

<u>Supp figure 3</u>: Flow cytometry gating strategy

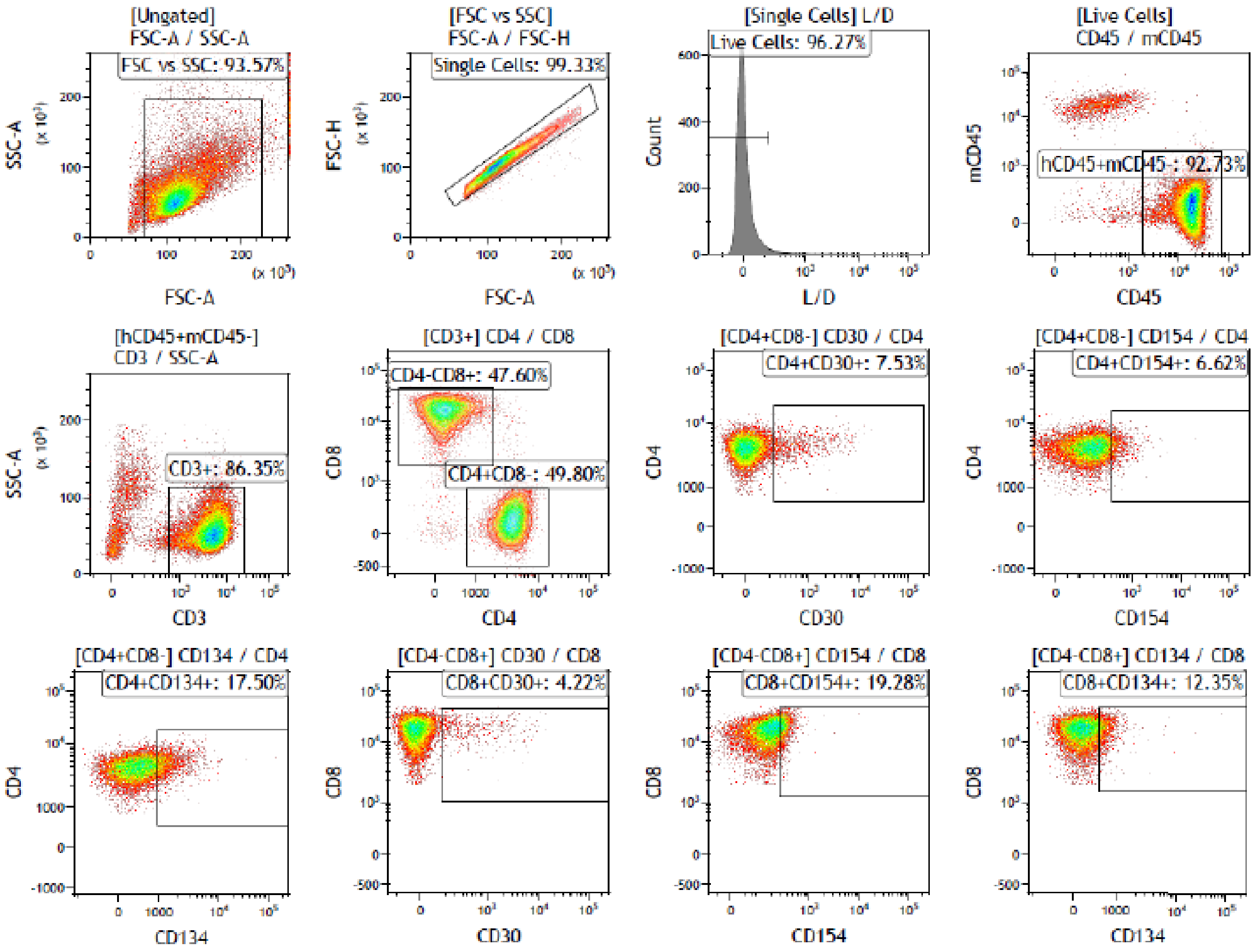

Representative flow cytometry plots and gating strategy of a terminal blood sample from a mouse in the MEDI8500 group.

<u>Supp figure 4</u>: Body weight changes

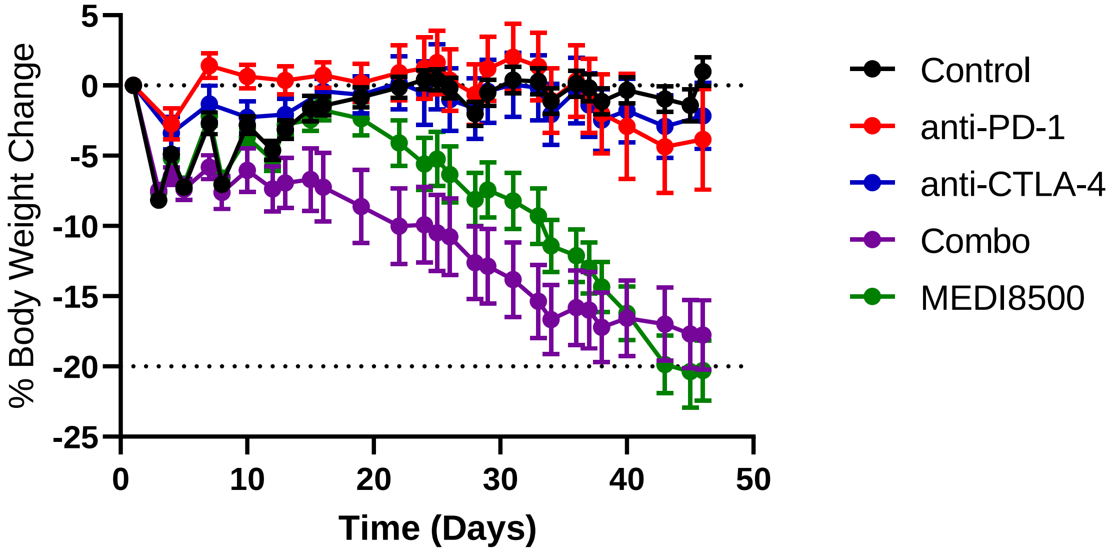

Mean percent body weight change ± SEM from all donors in the different treatment groups over time. Study day 1 corresponds to day of randomization and start of dosing. Mice numbers are the same as in Figure 2a.

Supp Figure 5: Increase of circulating human leucocytes in mouse blood after treatment

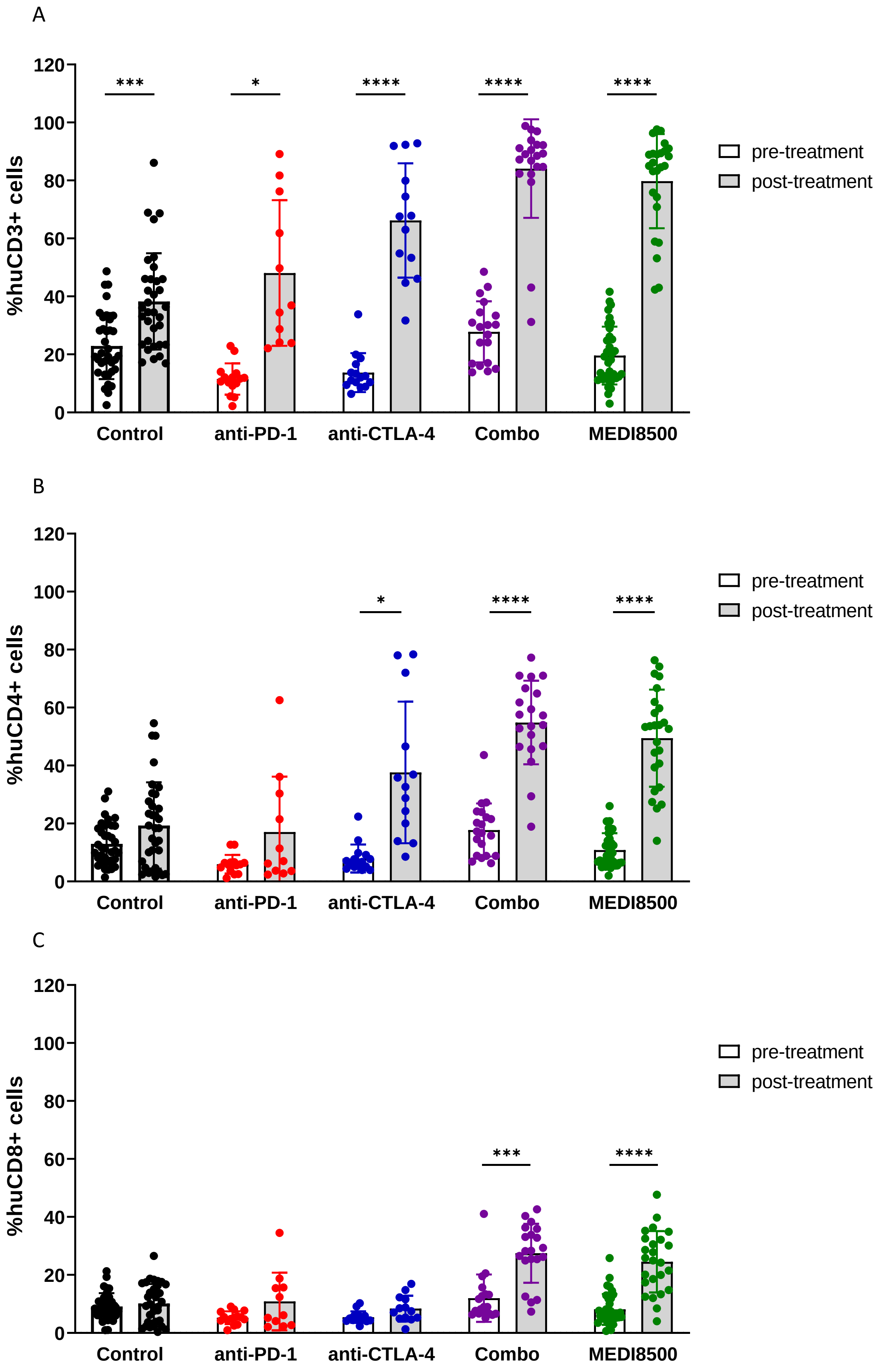

Flow cytometry analysis of the percentage of huCD45^{+} cells in blood of mice in the different groups of treatment. Data are represented as percentage within live population for each individual mouse and as Mean±SD of all mice in each treatment group. Mice numbers are the same as in Figure 2a. A Mixed-effects analysis followed by Sidak's multiple comparisons test was used to evaluate statistical differences between pre-treatment and post-treatment groups; *P < 0.05, ***P < 0.001.

<u>Supp Figure 6</u>: Comparison of huCD45 staining in tissues with or without GvHD

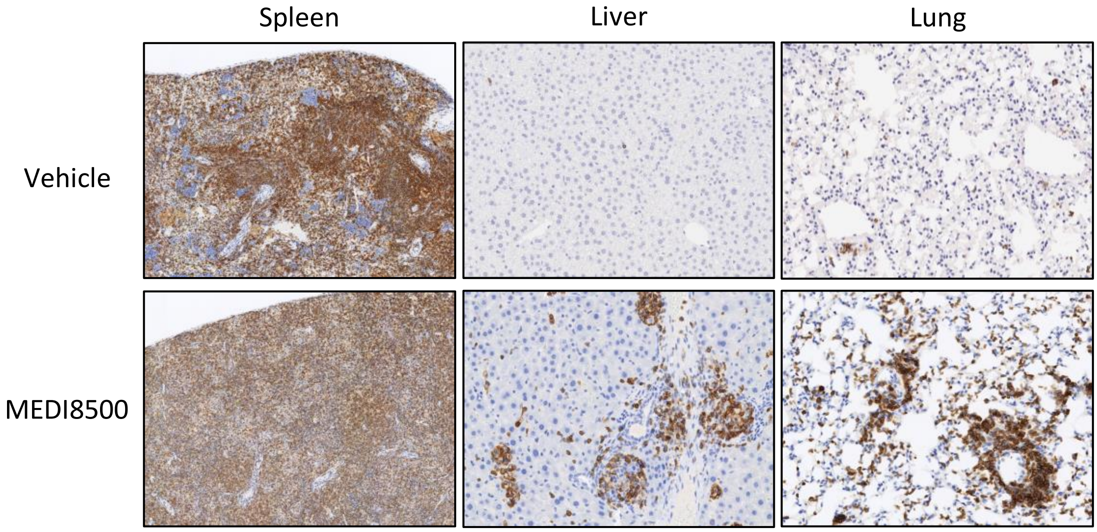

Representative images of huCD45 staining in spleen (10x magnification), liver (20x magnification) and lung (20x magnification) in one mouse in the vehicle group and one mouse in the MEDI8500 treated group
