# Supplementary material for: Characterization of a novel potency endpoint for the evaluation of immune checkpoint blockade in humanized mice
Source: Front Immunol. 2023 Mar 3;14:1107848. doi: 10.3389/fimmu.2023.1107848 (PMC10020612; doi:10.3389/fimmu.2023.1107848)
Supplement: Supplementary file 2 [file Table_1.docx]

**Supplementary Table 1**

|  | huCD45^+^ area% | | | |
| --- | --- | --- | --- | --- |
| Treatment |  | Liver | Lung | Spleen |
| Vehicle | | 0.8 ± 0.5 | 2.8 ± 1.4 | 28.7 ± 11.2 |
| anti-PD1 | | 3.5 ± 3.8 | 6.0 ± 5.0 | 37.9 ± 5.4 |
| anti-CTLA4 | | 3.1 ± 3.0 | 8.7 ± 4.9^a^ | 31.5 ± 7.3 |
| combo anti-PD1/CTLA4 | | 12.8 ± 5.8^b^ | 9.6 ± 7.2^a^ | 22.9 ± 7.3 |
| MEDI8500 | | 9.3 ± 6.9^b^ | 14.8 ± 6.3^c^ | 31.1 ± 8.3 |

**huCD45 quantitation results in tissues**: Data are percentage of huCD45 positive area as total tissue percentage, shown as mean ± SD per group. ^a^ significant difference (**P* < 0.05) with vehicle, ^b^ significant difference (**P* < 0.05) with vehicle, anti-PD-1 and anti-CTLA-4, ^c^ significant difference (**P* < 0.05) with vehicle, anti-PD-1, anti-CTLA-4 and combination anti-PD-1 + anti-CTLA-4.
